# Changes in lung mechanics and ventilation-perfusion match: comparison of pulmonary air- and thromboembolism in rats

**DOI:** 10.1186/s12890-024-02842-z

**Published:** 2024-01-10

**Authors:** József Tolnai, Bence Ballók, Roberta Südy, Álmos Schranc, Gabriella Varga, Barna Babik, Gergely H. Fodor, Ferenc Peták

**Affiliations:** 1 Department of Medical Physics and Informatics, University of Szeged, 9 Korányi fasor, Szeged, H-6720 Hungary; 2 Unit for Anesthesiological Investigations, Department of Anesthesiology, Pharmacology, Intensive Care and Emergency Medicine, University of Geneva, 1 Rue Michel-Servet, 1206 Geneva, Switzerland; 3 Institute of Surgical Research, University of Szeged, 1 Pulz utca, Szeged, H-6724 Hungary; 4 Department of Anesthesiology and Intensive Therapy, University of Szeged, 6 Semmelweis str., Szeged, H-6725 Hungary

**Keywords:** Pulmonary air embolism, Pulmonary artery occlusion, Respiratory mechanics, Arterial blood gas parameters, Volumetric capnography, Ventilation-perfusion mismatch

## Abstract

**Background:**

Pulmonary air embolism (AE) and thromboembolism lead to severe ventilation-perfusion defects. The spatial distribution of pulmonary perfusion dysfunctions differs substantially in the two pulmonary embolism pathologies, and the effects on respiratory mechanics, gas exchange, and ventilation-perfusion match have not been compared within a study. Therefore, we compared changes in indices reflecting airway and respiratory tissue mechanics, gas exchange, and capnography when pulmonary embolism was induced by venous injection of air as a model of gas embolism or by clamping the main pulmonary artery to mimic severe thromboembolism.

**Methods:**

Anesthetized and mechanically ventilated rats (*n* = 9) were measured under baseline conditions after inducing pulmonary AE by injecting 0.1 mL air into the femoral vein and after occluding the left pulmonary artery (LPAO). Changes in mechanical parameters were assessed by forced oscillations to measure airway resistance, lung tissue damping, and elastance. The arterial partial pressures of oxygen (PaO_2_) and carbon dioxide (PaCO_2_) were determined by blood gas analyses. Gas exchange indices were also assessed by measuring end-tidal CO_2_ concentration (ETCO_2_), shape factors, and dead space parameters by volumetric capnography.

**Results:**

In the presence of a uniform decrease in ETCO_2_ in the two embolism models, marked elevations in the bronchial tone and compromised lung tissue mechanics were noted after LPAO, whereas AE did not affect lung mechanics. Conversely, only AE deteriorated PaO_2_, and PaCO_2_, while LPAO did not affect these outcomes. Neither AE nor LPAO caused changes in the anatomical or physiological dead space, while both embolism models resulted in elevated alveolar dead space indices incorporating intrapulmonary shunting.

**Conclusions:**

Our findings indicate that severe focal hypocapnia following LPAO triggers bronchoconstriction redirecting airflow to well-perfused lung areas, thereby maintaining normal oxygenation, and the CO_2_ elimination ability of the lungs. However, hypocapnia in diffuse pulmonary perfusion after AE may not reach the threshold level to induce lung mechanical changes; thus, the compensatory mechanisms to match ventilation to perfusion are activated less effectively.

## Background

Pulmonary embolism is a common adverse cardiovascular event and can lead to severe gas exchange and circulatory consequences [1]. This life-threatening condition can occur when air or other gases enter the pulmonary circulation, which commonly occurs following lung trauma, as a complication of childbirth, or during surgical interventions [2], scuba diving, or flying [3]. Another form of pulmonary embolism occurs when the pulmonary vessels are occluded by a solid blood clot or other bodily substance, such as following deep venous thrombi or in cancer-associated thrombosis [1, 4].

Although both gas- and thromboembolism compromise pulmonary blood flow, these disorders differ substantially, particularly in the regional distribution of pulmonary perfusion defects. Gas bubbles in the venous blood undergo turbulent mixing and disruption in the right ventricle, mainly leading to gravitation-dependent diffuse pulmonary hypoperfusion [5–7]. Conversely, the spatial distribution of thromboembolism is more focal and mainly affects distinct lung areas [8–10]. These fundamental differences in the spatial distribution of the lung perfusion defects may affect the adverse changes in respiratory mechanics, gas exchange, and ventilation-perfusion match. Thus, major differences can be anticipated in the outcomes of these two forms of embolism. Nevertheless, the effects of pulmonary perfusion defects in models of thrombo- or gas embolism have not been compared in a study.

Therefore, we aimed to compare changes in indices reflecting airway and respiratory tissue mechanics, gas exchange, and capnography parameters when lung embolism is induced by venous injection of air bubbles as a model of gas embolism or by clamping the main pulmonary artery to mimic severe thromboembolism.

## Methods

### Ethical statement

This experimental protocol was included in a research project approved by the National Food Chain Safety and Animal Health Directorate of Csongrád County, Hungary (no. XXXII./2096/2018) on September 24, 2018. The experimental procedures were performed according to the guidelines of the Scientific Committee of Animal Experimentation of the Hungarian Academy of Sciences (updated Law and Regulations on Animal Protection: 40/2013. [II. 14.], Government of Hungary) and European Union Directive 2010/63/EU on the protection of animals used for scientific purposes. All methods are reported in accordance with ARRIVE guidelines for the reporting of animal experiments [11].

### Animal preparation

Nine male Wistar rats (362 ± 25 g) were anesthetized by the intraperitoneal injection of sodium pentobarbital (45 mg/kg; Sigma-Aldrich, Budapest, Hungary). The left femoral artery and vein were cannulated by 23 G catheters to administer medications, monitor blood pressure, and collect blood samples. Anesthesia was maintained with intravenous sodium pentobarbital (5 mg/kg) every 30 min, and muscle relaxation was obtained by neuromuscular blockade with repeated intravenous boluses of pipecuronium (0.2 mg/kg every 30 min; Arduan, Richter-Gedeon, Budapest, Hungary). The body temperature of the rats was maintained at 37 °C ± 0.5 °C throughout the experiment using a heating pad equipped with a rectal thermometer (model 507223F; Harvard Apparatus, Holliston, MA, USA).

The animals were tracheostomized after additional local anesthesia with subcutaneous lidocaine (2–4 mg/kg), and the trachea was cannulated with an 18 G uncuffed endotracheal tube. Volume-controlled mechanical ventilation with a tidal volume (VT) of 7 mL/kg and positive end-expiratory pressure (PEEP) of 5 cmH_2_O was applied using a small animal ventilator (Model 683; Harvard Apparatus, South Natick, MA, USA). The ventilation frequency was set to 55–60 breaths/min to maintain an end-tidal CO_2_ (ETCO_2_) level within the normal range (35–45 mmHg). Midline thoracotomy was then performed to access the left pulmonary artery. The mean arterial pressure (MAP) and heart rate (HR) were monitored and recorded using a data collection and acquisition system (Powerlab 8/35 and Labchart, ADInstruments, Dunedin, New Zealand).

### Measurement of airway and respiratory tissue mechanics

The mechanical parameters of the airway and respiratory tissues were determined by measuring the input impedance of the respiratory system (Zrs) using the wave-tube approach of the forced oscillation technique [12]. Briefly, the tracheal cannula was attached to a loudspeaker-in-box system, which generated a small-amplitude pseudorandom forcing signal (amplitudes < 1.5 cmH_2_O with 23 non-integer multiple-frequency components in the range of 0.5–20.75 Hz) through a wave-tube (polyethylene; length, 100 cm; internal diameter, 2 mm). During these measurements, ventilation was briefly suspended (8 s) at end-expiration, and the oscillatory pressures were measured with two identical miniature differential pressure transducers (Honeywell Differential Pressure Sensor model 24PCEFA6D; Honeywell, Charlotte, NC, USA) at the loudspeaker and tracheal ends of the wave-tube. After a minimum of four technically acceptable measurements in each protocol phase, Zrs values were calculated as the load impedance of the wave-tube [12]. The mechanical properties of the respiratory system were obtained by fitting the well-established and validated constant phase model to the ensemble-averaged Zrs spectra (16). This model comprised frequency-independent airway resistance (Raw) and airway inertance in series with a viscoelastic constant phase tissue unit that includes tissue elastance (H) and tissue damping (G) [13]. Hysteresivity, reflecting the coupling of the resistive and elastic properties within the viscoelastic respiratory tissues, was calculated as η = G/H [14].

### Blood gas analyses

Samples of 0.15 mL arterial blood were collected for blood gas analyses, from which the arterial partial pressures of oxygen (PaO_2_) and carbon dioxide (PaCO_2_) were determined using a point-of-care blood analyzer system (Epoc Reader and Host; Epocal, Inc., Ottawa, ON, Canada).

### Recording and analyses of volumetric capnograms

The partial pressure of carbon dioxide (PCO_2_) in expired gas and ventilation airflow (V′) were simultaneously recorded using a rodent sidestream volumetric capnograph (Harvard Capnograph Type 340 for small rodents) at a sampling frequency of 256 Hz. Volumetric capnogram curves were generated for each expiratory cycle from the PCO_2_ tracing and the volume signals obtained by integrating the corresponding V′ data. The capnogram phase boundaries were determined, and the shape factors were calculated based on previously described concepts [15, 16]. Briefly, the inflection point of phase 2 was determined as the maximum of the first derivative of the volumetric capnogram curve, and the phase 2 slope (S2V) was measured as the rate of change of PCO_2_ around this inflection point. The start and end points of phase 2, which reflect the mixed emptying of airway-alveolar spaces, were identified as the maxima of the third derivative before and after this inflection point in both the time and volumetric domains. The phase 3 capnogram slope (S3V), reflecting the dynamics of the alveolar gas compartment emptying, was determined by fitting a linear regression line to the middle third of phase 3. Normalized phase 2 (Sn2V) and 3 (Sn3V) slopes were calculated by dividing S2V and S3V by the respective ETCO_2_ values. This normalization allows a more objective comparison of changes in the capnogram shape due to changes in ETCO_2_, such as observed in the embolism models used in the present study [17–19].

Volumetric capnography also enables the calculation of Fowler’s anatomic dead space (VDF), Bohr’s physiological dead space (VDB), and Enghoff’s modified physiological dead space (VDE). VDF was defined as the exhaled gas volume until the inflection point in phase 2 [20]. VDB was calculated as [21]:

$$\text{VDB}/\text{VT}=\left({\text{PACO}}_2-\mathrm P\overline{\mathrm E}{\mathrm{CO}}_2\right)/{\text{PACO}}_2$$

where the mean alveolar partial pressure of CO_2_ (PACO_2_) was defined as the CO_2_ concentration at the midpoint of phase 3 in the volumetric capnography curve, and the mixed expired CO_2_ partial pressure (PĒCO_2_) was estimated by dividing the integrated capnogram curve by VT in each expiratory cycle.

VDE, which also comprises nonventilated but perfused alveoli, also known as intrapulmonary shunting, was obtained as follows [22, 23]:

$$\text{VDE}/\text{VT}=\left({\text{PaCO}}_2-\mathrm P\overline{\mathrm E}{\mathrm{CO}}_2\right)/{\text{PaCO}}_2$$

where PaCO_2_ is the partial pressure of CO_2_ in an arterial blood sample.

The alveolar dead space fraction (AVDSf) was also determined as (PaCO_2_-ETCO_2_)/PaCO_2_ [24].

### Pilot experiments

In these preliminary experiments, 4 and 2 animals were used to examine the hemodynamic and respiratory responses to air injections of 0.2 ml and 0.15 ml, respectively. However, the responses observed in these pilot rats were excessively severe, resulting in lethal outcomes for 3 rats. These preliminary findings were the basis of the decision to use a 0.1 ml air bubble in the main protocol group.

### Study protocol

Following anesthesia induction and surgical preparations, including the thoracotomy, the rats were ventilated as described above with a PEEP of 5 cmH_2_O throughout the study. After stabilizing vital parameters, a lung recruitment maneuver was performed by closing the expiratory limb of the ventilator tubing until the next expiration to standardize lung volume history. Baseline data were collected 1–2 minutes later by recording capnograph curves and forced oscillation data, along with blood gas analyses of arterial blood samples. Pulmonary air embolism (AE) was then induced by injecting 0.1 mL of air (0.5% of total blood volume) mixed with 0.9 mL of saline into the femoral vein while continuously monitoring the changes in systemic hemodynamics and the capnogram. This injected air volume was titrated in pilot experiments to exert similar changes in ETCO_2_ compared to those observed after unilateral pulmonary artery occlusion, yielding comparable gas exchange defects assessed in the expired gas (*see above*). When a peak decrease in ETCO_2_ was observed (10–15 seconds after the air injection), an additional data set was recorded (condition AE). The effects of AE on systemic hemodynamics and gas exchange parameters lasted approximately 2–4 minutes and then returned to the normal range after approximately 5 minutes, as verified by the continuous monitoring of relevant vital signs. After a 10–15-min stabilization period, the left pulmonary artery was clamped using a small-sized surgical clip, and a third data set, including capnography, forced oscillations, and blood gas analyses, was collected under this condition, i.e. during left pulmonary artery occlusion (LPAO).

### Statistical analysis

Data are presented as the median with interquartile ranges in each boxplot chart. The Shapiro–Wilk test was used to evaluate the data distributions for normality. For each parameter studied, one-way repeated-measures ANOVA with Holm–Šidák post hoc tests was applied to assess significant differences between the results obtained in the protocol phases. Statistical analyses were performed with the SigmaPlot software package (Version 14, Systat Software, Inc., Chicago, IL, USA).

Since changes in lung tissue stiffness were anticipated after embolism, H was considered the primary outcome variable for estimating sample size using one-way repeated-measures ANOVA, with an effect size (f) of 0.4, power of 0.8, and significance level of 0.05. This analysis performed using G*Power (version 3.1.9.7, Universität Düsseldorf, Germany) indicated that at least nine animals were required to detect a 15% significant difference in the primary outcome when considering the previously observed variability [25]. Statistical analyses were performed at a significance level of *P* < 0.05.

## Results

**Fig. 1:**
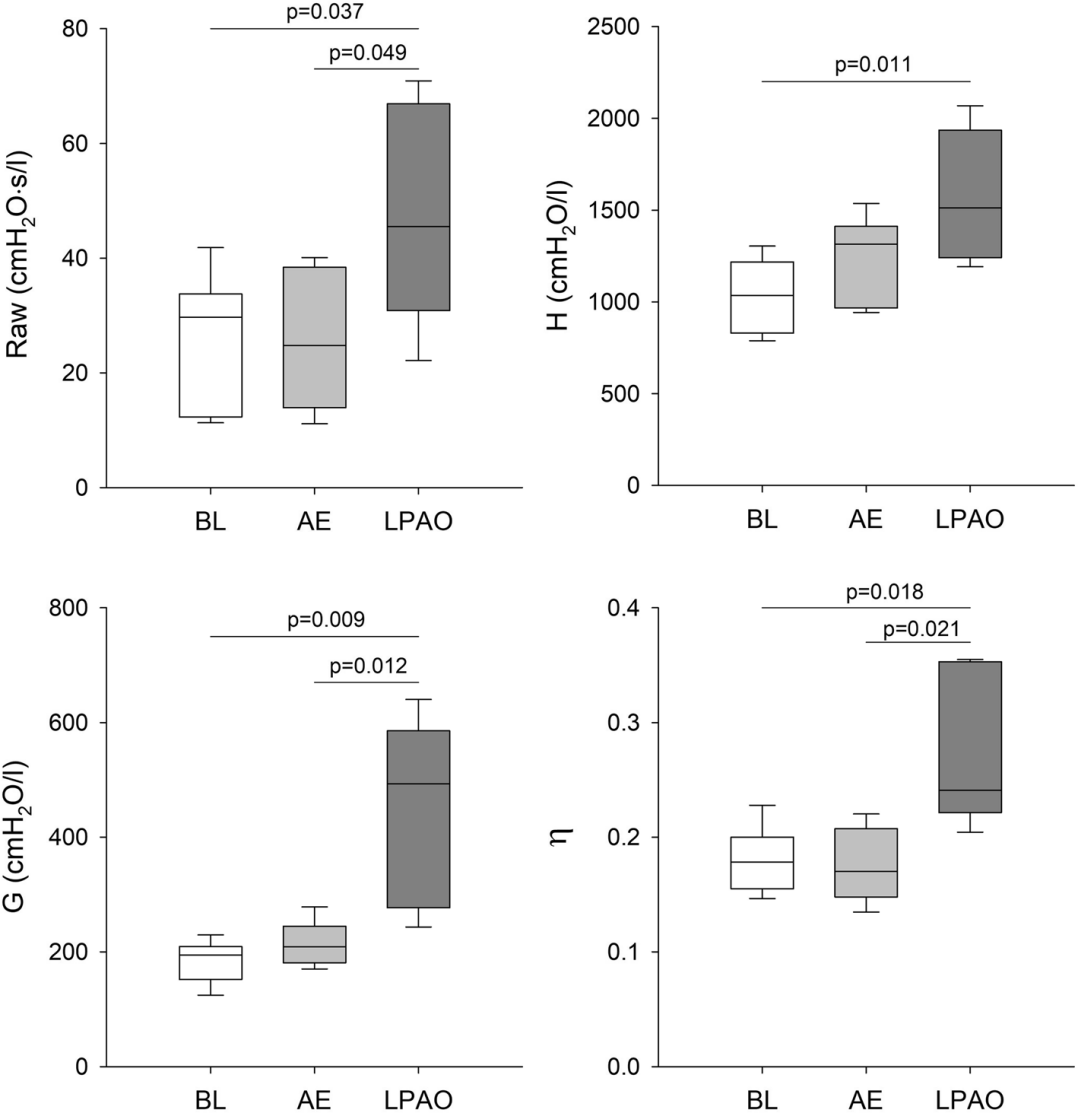
Airway and respiratory tissue mechanical parameters at baseline (BL), following air embolism (AE), and after left pulmonary artery occlusion (LPAO). Raw: airway resistance, H: tissue elastance, G: tissue damping, η: tissue hysteresivity (G/H)

The mechanical parameters of the respiratory system under baseline conditions and following induction of pulmonary embolism by two different mechanisms are shown in Fig. 1. No significant changes in these parameters were observed after AE. In contrast, significant increases in all parameters reflecting airway (Raw) and respiratory tissue mechanics (H, G, and η) were obtained after clamping the left pulmonary artery compared to baseline values (*P* < 0.05 for all). Significant differences in Raw, G, and η were observed between the two forms of embolism (*P* < 0.05 for all), while there was a trend toward a significant difference in H (*P* = 0.1).

**Fig. 2:**
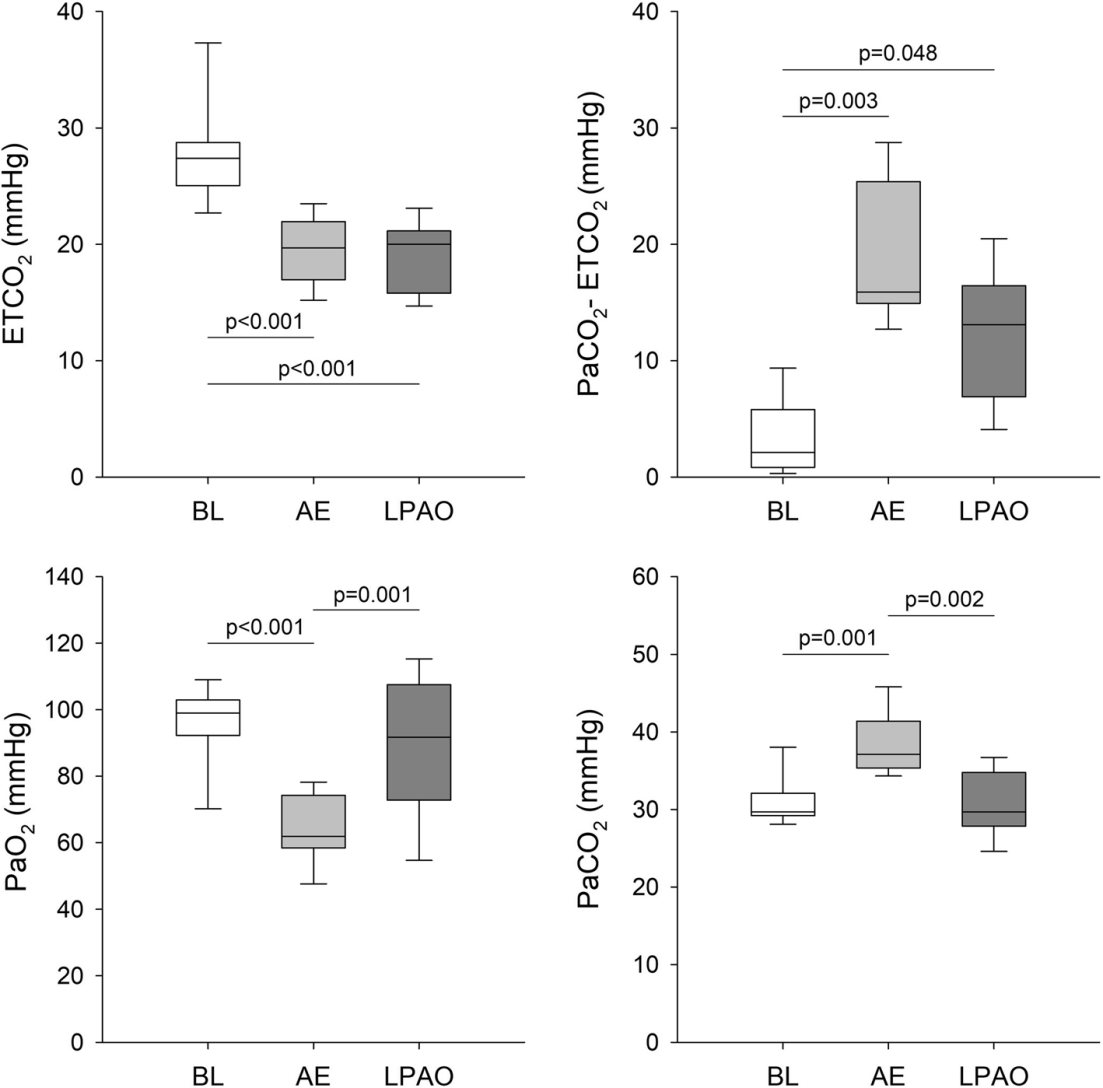
Arterial blood gas parameters and related capnogram indices obtained at baseline (BL), following air embolism (AE), and after left pulmonary artery occlusion (LPAO).ETCO_2_: end-tidal carbon dioxide, PaCO_2_-ETCO_2_: arterial to the end-tidal CO_2_ pressure gradient, PaO_2_: arterial partial pressure of oxygen, PaCO_2_: arterial partial pressure of carbon dioxide

Figure 2 summarizes ETCO_2_, the PaCO_2_-ETCO_2_ gradient, and the arterial blood gas parameters under the baseline condition and following the induction of embolism by the two different mechanisms. In accordance with our experimental approach to induce comparable gas exchange defects assessed in the expired CO_2_ concentration, the significant decreases in ETCO_2_ did not differ between the two embolism models (*p* < 0.001 vs. baseline for both). Furthermore, increases in PaCO_2_-ETCO_2_ of comparable magnitude were observed in both embolism models (*P* < 0.05 vs. baseline for both). A significant decrease in PaO_2_ and a significant increase in PaCO_2_ were observed following AE (*P* < 0.001 for both). However, these parameters were not significantly affected by LPAO, resulting in significant differences in PaO_2_ and PaCO_2_ between the two forms of pulmonary embolism (*P* < 0.001 for both). There was a strong tendency for an increase in the arterial lactate level, with increasing from the baseline value of 2.6 ± 0.8 mmol/L to 3.6 ± 1.3 and 4.3 ± 1.8 mmol/L after AE and LPAO, respectively (*p* = 0.056).

**Fig. 3:**
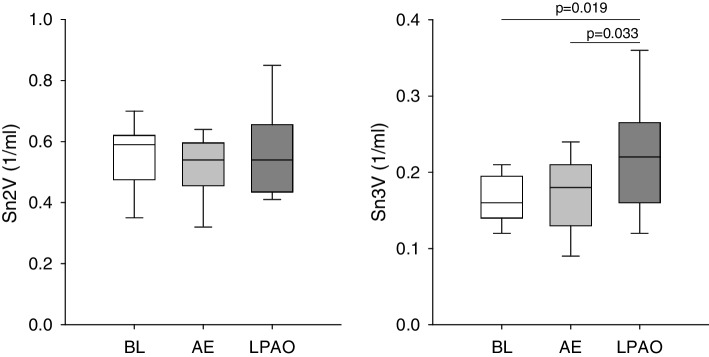
Phase 2 and 3 slopes of the volumetric capnogram normalized to the end-tidal CO_2_ concentration obtained at baseline (BL), following air embolism (AE), and after left pulmonary artery occlusion (LPAO)

Figure 3 shows shape factors representing the normalized phase 2 and phase 3 slopes obtained by volumetric capnography. No significant change in Sn2V was observed after either form of embolism. In contrast, significant increases in Sn3V were evidenced following LPAO compared to baseline and after AE (*P* < 0.05 for both).

**Fig. 4:**
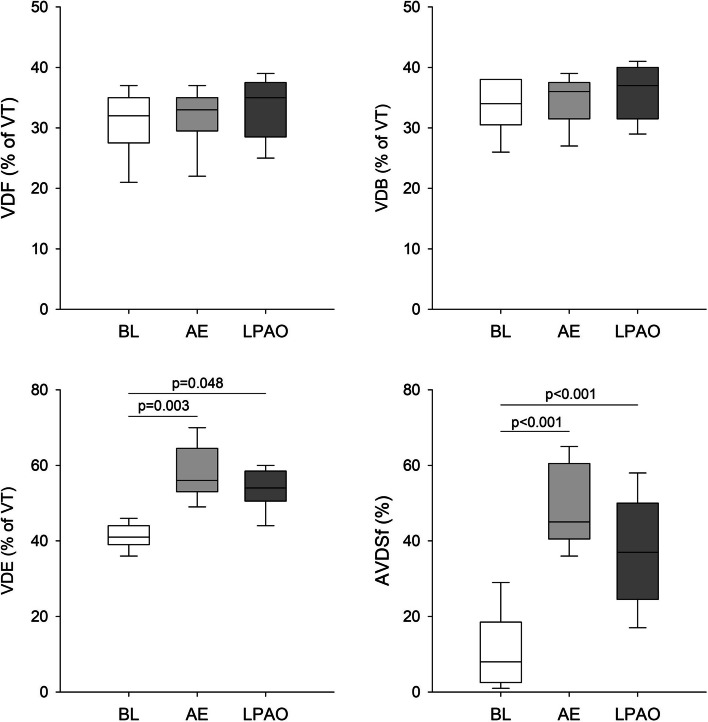
Fowler anatomical (VDF), Bohr (VDB), and Enghoff (VDE) physiological dead spaces, and alveolar dead space fraction (AVDSf) expressed as a percentage of tidal volume (% of VT) obtained at baseline (BL), following air embolism (AE), and after left pulmonary artery occlusion (LPAO)

Changes in dead space indices at different protocol stages are shown in Fig. 4. There were no significant changes in VDF or VDB following either form of embolism. However, a significant increase in the VDE dead space fraction was observed following AE and LPAO (*P* = 0.003 and *P* < 0.05, respectively). A significant increase in AVDSf compared to the baseline condition was also observed following both forms of embolism (*P* < 0.001 for both), with a trend toward increased AVDSf following AE compared to LPAO (*P* = 0.063).

Table 1 demonstrates the changes in the systemic hemodynamics before, during, and after the embolic insults. AE and LPAO induced significant transient decreases in MAP and HR (*p* < 0.001, for all). Complete recoveries of MAP and HR were observed after AE with no significant difference compared to the values obtained before the interventions.

**Table 1:** Mean arterial pressure (MAP) and heart rate (HR) expressed as mean ± SD obtained before air embolism (Before AE), at the peak response following AE, and 2 minutes later. Values are also reported immediately before the left pulmonary artery occlusion (LPAO), at the peak response after LPAO, and 2 minutes after release of the left pulmonary artery. *: *p* < 0.001 vs. BL, #: *p* < 0.001 vs. AE, $: *p* = 0.013 vs. AE

	Before AE	AE	2 mins after AE	Before LPAO	LPAO	2 mins after LPAO
MAP (mmHg)	116 ± 11	64 ± 21*	110 ± 9	125 ± 4	30 ± 10*^#^	108 ± 12
HR (BPM)	490 ± 57	352 ± 48*	474 ± 69	473 ± 24	215 ± 46*^$^	463 ± 24

## Discussion

The present study revealed fundamental differences between models of pulmonary embolisms, although comparable decreases in the expired carbon dioxide concentration were induced by the gas- and thromboembolic models. Unilateral pulmonary arterial occlusion caused marked elevations in the bronchial tone and compromised lung tissue mechanics, whereas air embolism did not affect lung mechanics. In contrast with these lung mechanical changes, air embolism was the only insult that caused significant deteriorations in the arterial blood gas parameters, while partial pressures of oxygen and carbon dioxide in the arterial blood were unaffected by left pulmonary artery occlusion. No changes in anatomical (Fowler) or physiological dead space reflecting ventilated but poorly perfused lung areas (Bohr) were observed in either embolism model. Conversely, both air embolism and unilateral pulmonary artery occlusion resulted in elevated alveolar dead space indices that indicate intrapulmonary shunting (Enghoff and AVDSf).

The main finding of the present study was that distinct lung mechanical responses developed in response to the different models of lung embolism, even in the presence of similarly deteriorated ETCO_2_ levels. In contrast to the lack of changes in lung mechanics following AE, marked bronchoconstriction developed in response to LPAO (Fig. 1). This finding can be explained by the ability of the CO_2_ content of the intrapulmonary gas to modulate the airway smooth muscle tone, resulting in severe bronchospasm below a threshold partial pressure of CO_2_ of approximately 10 mmHg [26]. Reaching this threshold CO_2_ concentration in the ischemic left lung during LPAO explains the elevations in airway resistance. The magnitude of the overall increase in the airway resistance was blunted by the intact right lung, which was probably somewhat overinflated. Conversely, the diffuse pulmonary hypoperfusion that developed after AE was likely to cause a moderate and relatively evenly distributed hypocapnia in the bronchoalveolar system, in which the threshold concentration to trigger a bronchial smooth muscle contraction was not reached [26].

These differences in the airway responses between AE and LPAO were associated with distinct changes in the forced oscillatory parameters reflecting respiratory tissue damping (G) and stiffness (H). The excessive increase in G over those in H indicates the development of ventilation heterogeneities [27]. As this pattern of change was observed only after LPAO, the impairment in respiratory tissue mechanics indicates that considerable ventilation inhomogeneity developed after LPAO due to uneven lung ventilation resulting from the additive effects of volume loss in the left lung and overdistension of the right lung. The presence of this phenomenon was confirmed by the elevations in mechanical and capnography parameters sensitive to ventilation heterogeneities (η and Sn3V).

Changes in blood gas parameters notably differed between the two embolism models, with significant deteriorations observed only after a diffuse pulmonary perfusion defect generated by AE (Fig. 2). In agreement with previous findings [28–30], acute AE led to the development of hypoxia and hypercapnia. The adverse gas exchange defects were reflected in the alveolo-end-tidal CO_2_ gradient (Fig. 2) and in the marked elevations in VDE and AVDSf (Fig. 4). Since VDE and AVDSf incorporate poorly ventilated but perfused alveoli with low V/Q [31], increased intrapulmonary shunting may be responsible for this finding as a consequence of a perfusion redistribution [30]. Interestingly, blood gas parameters were unaffected by LPAO despite concomitant increases in capnography parameters reflecting intrapulmonary low V/Q areas and shunting (PaCO_2_-ETCO_2_ in Fig. 2 and VDE and AVDSf in Fig. 3) and in the airway and respiratory tissue mechanics (Fig. 1). The absence of changes in PaO_2_ following LPAO agrees with previous results demonstrating that oxygenation can remain normal following acute pulmonary thromboembolism [32, 33]. This finding is likely attributable to the unaffected right lung being able to maintain blood gas parameters within the normal range due to increased VT in response to hypocapnic bronchoconstriction in the ischemic left lung [34].

No change in VDF was observed after inducing lung embolism using air bubbles or unilateral pulmonary vascular ischemia (Fig. 3). As VDF reflects the amount of gas in the conducting airways [20], this finding demonstrates that there was no change in the anatomical dead space fraction following either intervention. While severe bronchoconstriction in the hypocapnic left lung may yield a unilateral reduction in apparent anatomic dead space [35], the redistribution of tidal volume to the right lung may have overinflated this compartment, thereby counterbalancing the anatomic dead space.

The lack of change in VDB after either form of embolism is worth noting (Fig. 3). Unilateral occlusion of the pulmonary artery is expected to result in nonperfused but well-ventilated lung regions. Furthermore, the development of a similar ventilation-perfusion mismatch is expected after introducing air into the pulmonary circulation. Accordingly, elevations in VDB can be anticipated in both models of embolism based on Riley’s 3-compartment lung model, in which VDB reflects the amount of well-ventilated but poorly perfused alveolar compartments [36]. This seemingly controversial result can be explained by the complex pathophysiological processes initiated by the massive ventilation-perfusion mismatch after embolism. Development of severe bronchoconstriction in the hypocapnic left lung following LPAO can reach a degree where airflow redistribution from the left into the right lung results in a constant VDB. The presence of this mechanical defect after LPAO was confirmed by our forced oscillatory data demonstrating marked elevations in airway resistance (Fig. 1). The lack of change in VDB following AE may be attributable to the relatively small amount of air reaching the pulmonary circulation (6–8% of total intrapulmonary blood volume) that may have caused no major perfusion defects.

A methodological consideration of the present protocol is related to the protocol design, which focused on the acute consequences of pulmonary embolism. This approach allowed the comparison of two pulmonary circulatory insults in an animal model, thereby strengthening the statistical power and reducing the need for an excessive number of animals in accordance with the 3R principle. Indeed, complete recovery of monitored gas exchange (ETCO_2_) and systemic circulatory parameters (MAP, HR) was observed between the interventions (Table 1), which suggests the lack of a carryover effect between the protocol stages. However, the generalization of our findings to prolonged pulmonary perfusion defects should be considered. An additional limitation of the present study is that volumetric capnography provides an overall picture about the V/Q matching for the entire lungs. Expanded use of imaging techniques, such as SPECT [37], K-edge subtraction [38], electric impedance tomography [39], or quantitative V/Q analysis by inert gas elimination [40] have the potential in forthcoming studies to further investigate the mechanisms by allowing regional assessments.

## Conclusions

Marked differences in lung mechanical, gas-exchange, and capnography indices were observed following diffuse lung injury induced by the intravenous injection of air bubbles as a model of gas embolism or following clamping of the left pulmonary artery to mimic focal thromboembolism. Deteriorations in the lung mechanics reflecting the development of heterogeneous bronchoconstriction were observed after a focal ischemic insult in the pulmonary perfusion. However, the severity of hypocapnia did not reach the threshold level to alter the bronchial tone in any lung regions following a diffuse pulmonary perfusion defect subsequent to air embolism. In contrast with these lung mechanical changes, arterial blood gas parameters deteriorated following a diffuse pulmonary perfusion defect due to air embolisms, while focal perfusion defects due to pulmonary vascular occlusion triggered a compensatory respiratory mechanical response leading to airflow redistribution to lung areas with maintained perfusion, thereby maintaining normal oxygenation and CO_2_ elimination. The findings that air and thromboembolism affect lung mechanics and gas exchange differently may have a relevance in tailoring respiratory support for patients.

## Data Availability

The datasets used and/or analysed during the current study are available from the corresponding author on reasonable request.

## Abbreviations

AE: Air embolism
ANOVA: Analysis of variance
ARRIVE: Animal research: reporting of in vivo experiments
AVDSf: Alveolar dead space fraction
ETCO_2_: End-tidal carbon dioxide concentration
G: Tissue damping
H: Tissue elastance
HR: Heart rate﻿
LPAO: Left pulmonary artery occlusion
MAP: Mean arterial pressure
PaO_2_: Arterial partial pressures of oxygen
PaCO_2_: Arterial partial pressures of carbon dioxide
PACO_2_: Mean alveolar partial pressure of carbon dioxide
PCO_2_: Partial pressure of carbon dioxide
PEEP: Positive end-expiratory pressure
PĒCO_2_: Mixed expired CO_2_ partial pressure
Raw: Airway resistance
S2V: Phase 2 slope of the volumetric capnogram
S3V: Phase 3 slope of the volumetric capnogram
Sn2V: Normalized phase 2 slope of the volumetric capnogram
Sn3V: Normalized phase 3 slope of the volumetric capnogram
V′: Ventilation airflow
VT: Tidal volume
VDB: Bohr’s physiological dead space
VDE: Enghoff’s modified physiological dead space
VDF: Fowler’s anatomic dead space
Zrs: Input impedance of the respiratory system
η: Tissue hysteresivity
